# Sequence Features and Transcriptional Stalling within Centromere DNA Promote Establishment of CENP-A Chromatin

**DOI:** 10.1371/journal.pgen.1004986

**Published:** 2015-03-04

**Authors:** Sandra Catania, Alison L. Pidoux, Robin C. Allshire

**Affiliations:** Wellcome Trust Centre for Cell Biology and Institute of Cell Biology, School of Biological Sciences, The University of Edinburgh, Edinburgh, United Kingdom; Netherlands Cancer Institute, NETHERLANDS

## Abstract

Centromere sequences are not conserved between species, and there is compelling evidence for epigenetic regulation of centromere identity, with location being dictated by the presence of chromatin containing the histone H3 variant CENP-A. Paradoxically, in most organisms CENP-A chromatin generally occurs on particular sequences. To investigate the contribution of primary DNA sequence to establishment of CENP-A chromatin *in vivo*, we utilised the fission yeast *Schizosaccharomyces pombe*. CENP-A^Cnp1^ chromatin is normally assembled on ∼10 kb of central domain DNA within these regional centromeres. We demonstrate that overproduction of *S*. *pombe* CENP-A^Cnp1^ bypasses the usual requirement for adjacent heterochromatin in establishing CENP-A^Cnp1^ chromatin, and show that central domain DNA is a preferred substrate for *de novo* establishment of CENP-A^Cnp1^ chromatin. When multimerised, a 2 kb sub-region can establish CENP-A^Cnp1^ chromatin and form functional centromeres. Randomization of the 2 kb sequence to generate a sequence that maintains AT content and predicted nucleosome positioning is unable to establish CENP-A^Cnp1^ chromatin. These analyses indicate that central domain DNA from fission yeast centromeres contains specific information that promotes CENP-A^Cnp1^ incorporation into chromatin. Numerous transcriptional start sites were detected on the forward and reverse strands within the functional 2 kb sub-region and active promoters were identified. RNAPII is enriched on central domain DNA in wild-type cells, but only low levels of transcripts are detected, consistent with RNAPII stalling during transcription of centromeric DNA. Cells lacking factors involved in restarting transcription—TFIIS and Ubp3—assemble CENP-A^Cnp1^ on central domain DNA when CENP-A^Cnp1^ is at wild-type levels, suggesting that persistent stalling of RNAPII on centromere DNA triggers chromatin remodelling events that deposit CENP-A^Cnp1^. Thus, sequence-encoded features of centromeric DNA create an environment of pervasive low quality RNAPII transcription that is an important determinant of CENP-A^Cnp1^ assembly. These observations emphasise roles for both genetic and epigenetic processes in centromere establishment.

## Author Summary

The kinetochore directs the separation of chromosomes and is assembled at a special region of the chromosome—the centromere. DNA is wrapped around particles called nucleosomes, which contain histone proteins. The nucleosomes at centromeres are specialized, and contain the centromere-specific histone CENP-A. CENP-A nucleosomes form the platform upon which the kinetochore is built. Thus, CENP-A and centromere function go hand-in-hand. How the cell ensures that CENP-A is deposited at centromeres and not elsewhere is not well understood. We investigated the role that DNA sequence plays in defining centromere function in fission yeast. Our observations suggest that it is not the DNA sequence per se that is important for attracting CENP-A, but rather, the particular environment that the sequence creates. During transcription of centromeric DNA, RNA polymerase (RNAPII) appears to get stuck or stalled. Particular proteins—such as TFIIS and Ubp3—are known to help restart RNAPII so it can continue transcribing. We found that when cells lack Ubp3 or TFIIS, CENP-A becomes deposited on centromere sequences. We propose that persistent stalling of RNAPII on centromere DNA attracts factors that help deposit CENP-A. This study highlights the influence of DNA sequence in creating an attractive environment for CENP-A assembly.

## Introduction

Centromeres are the chromosomal sites upon which kinetochores are assembled to ensure accurate segregation of sister chromatids into daughter cells. Most kinetochores are built upon a specialized type of chromatin in which canonical histone H3 is replaced by the histone variant CENP-A. Although the centromere-kinetochore complex performs conserved essential functions, and kinetochore proteins are generally conserved [1], centromeric DNA is not conserved, even between related species, and a huge variety of centromere sequences and structures exist [2–5]. The point centromeres of budding yeast consist of 125 bp of DNA and utilize an essential centromere-specific DNA binding protein [6]. At the other extreme, the nematode, *Caenorhabditis elegans*, has holocentric centromeres, in which kinetochore proteins assemble at multiple loci along each chromosome arm [7,8]. The majority of centromeres studied to date are regional. Centromeres in various plant and animal species are composed of arrays of different types of satellite, repetitive sequences and transposable elements, for instance, human centromeres encompass several megabases of tandem repetitive arrays of alpha-satellite sequence [9–11]. Fission yeast centromeres represent another type of regional centromere, in which a unique central core of 4–7 kb is flanked by inverted repeat elements and blocks of relatively large repeat units, resulting in centromeres of 40–120 kb [12]. Even the centromeres of different chromosomes in individual species are not necessarily homologous; each *Candida albicans* centromere has a unique central core, whilst chicken and potato each utilize both repeat-rich and unique sequence centromeres [13–15]. Thus, functional centromeres are assembled on diverse types of sequences in different organisms and it remains unknown if there is a universal fundamental property that defines centromeric sequences.

Abundant evidence indicates that centromeres are epigenetically regulated [16]. Although rare, neocentromeres have been observed in many species, forming on DNA sequences that do not normally possess centromere function and share no sequence homology with normal centromeres [17]. The best-characterized example in human is the neocentromere in 10q25 on the long arm of chromosome 10 that arose upon deletion of the centromere and loss of the entire alpha satellite array [18]. In *S*. *pombe*, neocentromeres form in close proximity to telomeres following the engineered deletion of a centromere [19]. Conversely, centromeres can be inactivated on dicentric human chromosomes despite the continued presence of alpha-satellite sequence at both centromeric loci [20]. In *S*. *pombe* one centromere on a dicentric chromosome can be inactivated by mechanisms such as heterochromatinisation or formation of a domain of histone hypoacetylation [21]. These and numerous other examples demonstrate that centromeric sequences are neither necessary nor sufficient for kinetochore assembly.

The histone H3 variant, CENP-A acts as the epigenetic mark that specifies centromere identity [22–24]. CENP-A is found only at active centromeres, including neocentromeres, and is absent at inactivated centromeres. The forced recruitment of CENP-A either by directly tethering CENP-A or its chaperone (HJURP) to a non-centromeric locus leads to the accumulation of CENP-A and kinetochore proteins at that location [24,25]. It is thought that continued deposition of CENP-A at centromere regions through cell and organism generations involves a self-propagation mechanism in which CENP-A chromatin, or features of the kinetochore which is assembled upon it, are recognized and attract additional CENP-A [26,27].

In most organisms there is no obligate coupling of sequence and CENP-A assembly, yet kinetochores are normally assembled upon particular centromeric sequences in any given species [4]. This suggests that centromeric sequences possess underlying properties that promote CENP-A incorporation. Alternatively, the preponderance of particular sequences at centromeres could be driven by properties of CENP-A chromatin or kinetochores themselves [28]. However, centromeric DNA allows the *de novo* assembly of functional centromeres following its introduction into cells in many organisms. Alpha satellite arrays are able to direct the *de novo* assembly of centromeres when introduced into certain cell lines as naked DNA [29,30]. *De novo* assembly of centromeres also occurs when centromeric DNA from *S*. *pombe* is introduced into cells. However, *de novo* establishment does not seem to be a universal property: despite promiscuous neocentromere formation in *C*. *albicans*, transformation with *bone fide* centromeric sequences does not result in kinetochore assembly[13]. At the other extreme, many sequences introduced into the holocentric organism *C*. *elegans* appear able to assemble CENP-A chromatin [31,32]. Thus, the relationship between centromeric sequence and the establishment and maintenance of CENP-A chromatin is enigmatic.

Transcription has received a lot of attention as a possible contributor to assembly of CENP-A chromatin. Transcripts emanating from centromeric regions have been detected in many organisms, including maize, human, rice, budding yeast, fission yeast and tammar wallaby [33–38]. Interfering with the chromatin status or transcriptional properties of centromeric repeats affects maintenance of CENP-A chromatin and segregation function on human artificial chromosomes (HACs) [39,40]. RNA Polymerase II (RNAPII) has been detected at mitotic mammalian centromeres where it may influence centromere function [38]. In fission yeast, transient H2B ubiquitylation may loosen centromeric chromatin to promote transcription and CENP-A^Cnp1^ incorporation and defective reassembly of H3 chromatin behind elongating RNAPII aids CENP-A^Cnp1^ incorporation [41,42]. However, although there are numerous tantalising hints that transcriptional activity contributes to centromere function or identity, much remains to be understood [33,38,43,44].

Here we investigate the contribution of DNA sequence to the establishment of CENP-A chromatin in fission yeast, an organism in which epigenetic mechanisms clearly influence centromere identity. Normally proximal heterochromatin is required to facilitate establishment of CENP-A^Cnp1^ chromatin on centromere central domain sequences [45,46]. We show that this requirement can be bypassed by overexpression of CENP-A^Cnp1^ and that central domain DNA is a preferred substrate for establishment of CENP-A^Cnp1^ chromatin. We find that there is functional redundancy within the central domain but that sub-regions are non-equivalent in their ability to establish CENP-A^Cnp1^ chromatin. Analysis of a 2 kb region capable of directing CENP-A^Cnp1^ assembly indicates that it contains numerous transcriptional start sites, along with promoter elements, and that relatively high levels of RNAPII are recruited, despite low levels of transcripts produced, consistent with the presence of stalled RNAPII. Our observations suggest that redundant sequence features in the centromere central domain create a unique transcriptional environment that is permissive for CENP-A^Cnp1^ establishment. Consistent with this, defective transcriptional elongation where stalled RNAPII is increased promotes the establishment of CENP-A^Cnp1^ chromatin.

## Results

### Elevated CENP-A^Cnp1^ levels bypass the requirement for heterochromatin in establishing CENP-A^Cnp1^ chromatin

**Fig 1:**
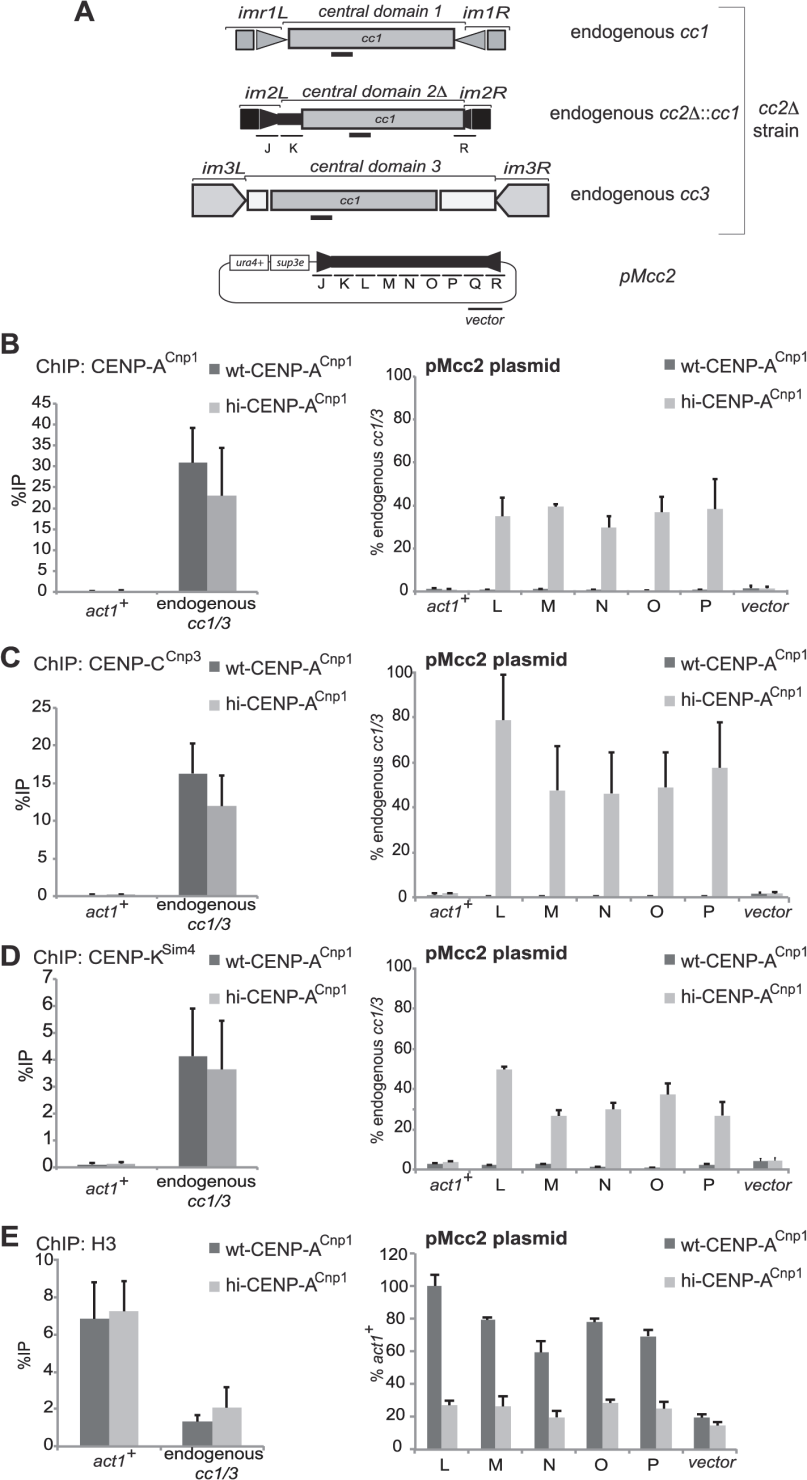
Elevated levels of CENP-A^Cnp1^ are sufficient to establish centromeric chromatin in the absence of heterochromatin. (A) Schematic representation of the three endogenous centromeres in *cc2Δ* strains and the plasmid (pMcc2) used in the study. In *cc2Δ* strains, part of the central core 2 (regions L to Q, each 1 kb) is replaced by 5.5 kb of DNA from central core 1, allowing the analysis of the L-Q sequence within pMcc2. Primer pairs used for quantification at endogenous centromeres recognise the region of homology shared between *cc1* and *cc3*. The product amplified by qPCR for the endogenous centromeres is represented as a black bar. (B) ChIP analysis of CENP-A^Cnp1^ levels at endogenous centromere (*cc1*/3), *act1* ^+^, plasmid backbone (vector) and at *cc2* on the plasmid (pMcc2: L-P) in wild-type (wt-CENP-A^Cnp1^) or in the presence of high levels of CENP-A^Cnp1^ (hi-CENP-A^Cnp1^). (C) ChIP analysis for the kinetochore protein CENP-C^Cnp3^. (D) ChIP analysis for the kinetochore protein CENP-K^Sim4^. (E) ChIP analysis of histone H3 levels. For the kinetochore proteins analysed, ChIPs are reported as %IP for endogenous centromeres or as relative to *cc1*/3 for the pMcc2 plasmid. For the levels of H3, the ChIP analyses are reported as %IP for *cc1*/3 and as relative to *act1* ^+^ for the pMcc2 plasmid (n = 3).

In wild-type fission yeast cells, *de novo* CENP-A^Cnp1^ chromatin establishment on circular plasmid-based minichromosomes requires an outer repeat or tethered Clr4 histone H3K9 methyltransferase to form a block of heterochromatin in close proximity to central domain DNA from centromeres [45,46]. CENP-A^Cnp1^ can also be deposited at other non-centromeric locations in the genome when it is overexpressed, however the level incorporated at these ectopic sites is much lower than that detected at natural centromeres [41,47]. To determine whether central domain DNA is a preferential substrate for the establishment of CENP-A^Cnp1^ chromatin, plasmid pMcc2 bearing 8.5 kb of central domain from *cen2* (*imr2-cc2-imr2*) sequence, but no heterochromatic outer repeat sequences, was transformed into cells expressing additional GFP-CENP-A^Cnp1^ (Fig. 1A).

All strains used have 6 kb of *cen2* central domain DNA replaced with 5.5 kb of *cen1* central domain DNA (*cc2Δ*::*cc1*—Fig. 1A, S1 Fig.) so that only 2.5 kb of normal *cen2* central domain DNA remains at this modified *cen2* (*imr2L*, regions J, K, R; Fig. 1A). The resulting deletion of fragments L-Q from the *cen2* central domain allows detailed and specific analysis of 6 kb of central domain DNA when borne by plasmid-based minichromosomes. Quantitative chromatin immunoprecipitation assays (qChIP) shows that CENP-A^Cnp1^ chromatin does not assemble on regions L, M N, O or P when a plasmid (pMcc2) containing the 8.5 kb *cc2* sequence, but lacking heterochromatin, was transformed into wild-type cells [45]. However, when pMcc2 was transformed into cells over-expressing CENP-A^Cnp1^ (hi-CENP-A^Cnp1^; ∼15 fold more than wild-type cells [41]), CENP-A^Cnp1^ and the kinetochore proteins CENP-C^Cnp3^ and CENP-K^Sim4^ were easily detected over the central domain of pMcc2 by qChIP (Fig. 1B-E). Importantly, these centromeric proteins were enriched on centromeric DNA but not on the plasmid backbone, indicating that CENP-A^Cnp1^ chromatin assembles specifically on central domain DNA from centromeres (Fig. 1). The relative level of enrichment of CENP-A^Cnp1^ and the other kinetochore proteins on different parts of pMcc2 suggests all proteins are distributed uniformly across this plasmid-borne central domain (Fig. 1B-D). Furthermore, the levels of histone H3 associated with the L-P regions of pMcc2 were reduced in cells expressing additional CENP-A^Cnp1^ compared to control cells (Fig. 1E). We conclude that H3 chromatin is normally assembled on central domain DNA on pMcc2 in wild-type cells but CENP-A^Cnp1^ chromatin assembles instead when pMcc2 is placed in hi-CENP-A^Cnp1^ cells.

### CENP-A^Cnp1^ can assemble on pre-chromatinised substrates and is trans-generationally inherited

**Fig 2:**
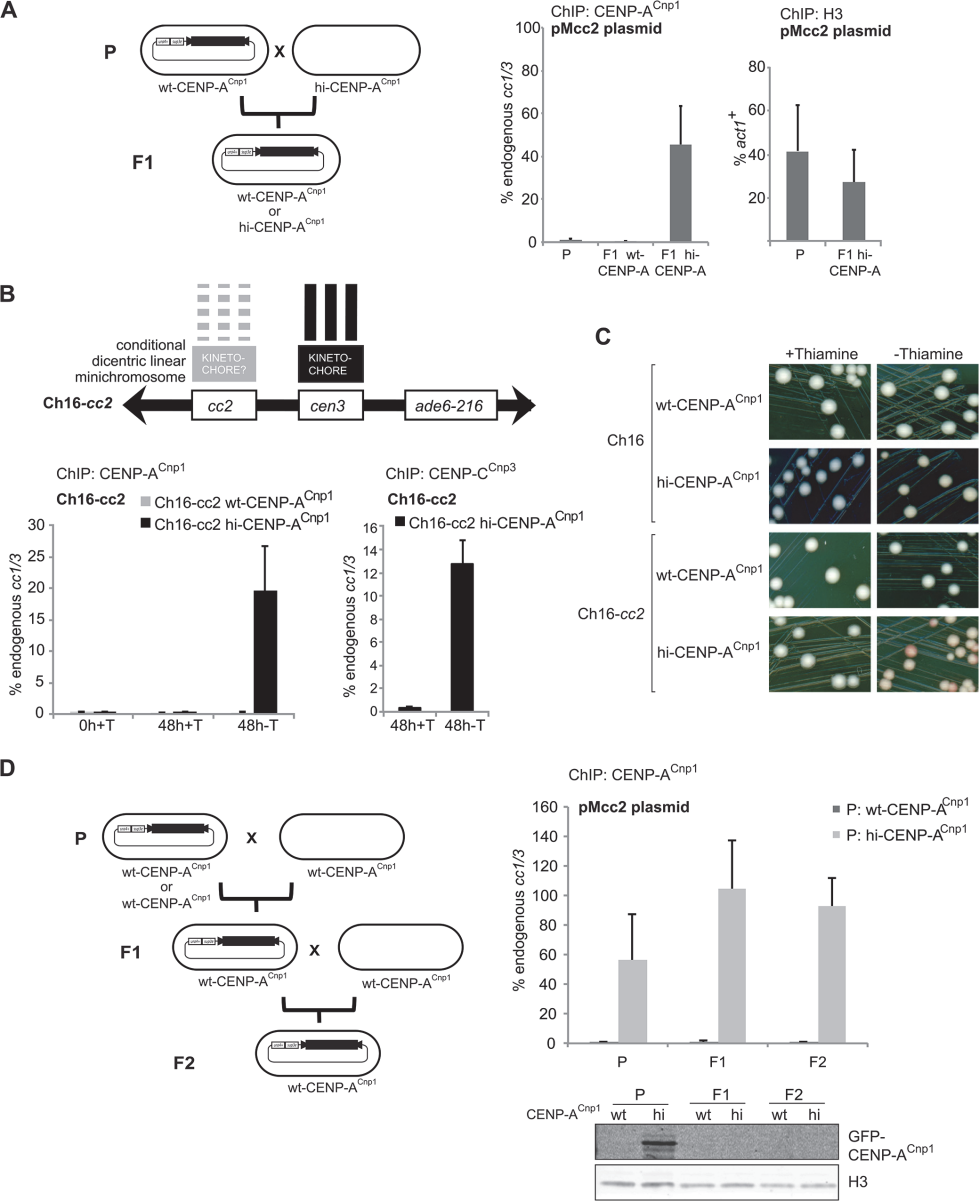
CENP-A^Cnp1^ chromatin behaves as an epigenetic mark. (A) A wild-type strain (parental: P; wt-CENP-A^Cnp1^) was transformed with the pMcc2 plasmid and then crossed to a strain overexpressing CENP-A^Cnp1^ (hi-CENP-A^Cnp1^). From the first generation (F1) cells containing pMcc2 and expressing wild-type level or overexpressing CENP-A^Cnp1^ were selected and analysed by ChIP for CENP-A^Cnp1^ and H3 levels on the pMcc2 plasmid (P: n = 2, F1: n = 4) (B) *cc2* was integrated on the arm of Ch16, that contains cen3, creating Ch16-*cc2*. Ch16-*cc2* was crossed into a strain containing nmt41-GFP-CENP-A^Cnp1^. In presence of thiamine (0h+T) GFP-CENP-A^Cnp1^ is repressed. The same cells were diluted in medium containing thiamine (48+T; GFP-CENP-A^Cnp1^ repressed) or without thiamine (48h-T; GFP-CENP-A^Cnp1^ expressed) for 48h. ChIP was performed for CENP-A^Cnp1^ and CENP-C^Cnp3^ levels. (C) Colony colour sectoring assay for cells containing minichromosome Ch16 or Ch16-*cc2*. Red sectors indicate loss of minichromosomes. (D) A wild-type strain (wt-CENP-A^Cnp1^) or a strain overexpressing CENP-A^Cnp1^ (hi-CENP-A^Cnp1^) were transformed with the pMcc2 plasmid (parental: P) and then crossed to a wt-CENP-A^Cnp1^ strain. From the first generation (F1), wt-CENP-A^Cnp1^ strains containing pMcc2 were selected and subsequently crossed to wt-CENP-A^Cnp1^ strain. Progeny resulting from this second cross are referred to as second generation (F2). ChIP analysis for CENP-A^Cnp1^ levels on the pMcc2 plasmid in the different generations relative to *cc1*/3; region M analysed by qPCR (P: n = 2, F1: n = 4, F2: n = 8).

To determine whether CENP-A^Cnp1^ can become established on plasmids that are already assembled in chromatin, the pMcc2 plasmid was transformed into cells expressing wt-CENP-A^Cnp1^ levels and subsequently crossed with hi-CENP-A^Cnp1^ cells. qChIP analyses indicate that CENP-A^Cnp1^ is initially absent from pMcc2 in the wt-CENP-A^Cnp1^ parental strain and then becomes assembled in CENP-A^Cnp1^ chromatin when transferred into the hi-CENP-A^Cnp1^ environment, indicating that plasmid-borne cc2 initially assembled in normal (H3) chromatin can be converted to CENP-A^Cnp1^ chromatin (Fig. 2A).

In addition, a copy of *cc2* (8.5 kb) was inserted on the arm of the 530 kb Ch16 linear minichromosome which carries a complete *cen3* [48] (Ch16-*cc2;* Fig. 2B). When the expression of additional GFP-CENP-A^Cnp1^ was repressed (0h+T), no CENP-A^Cnp1^ was detected on *cc2*. However, when GFP-CENP-A^Cnp1^ was induced (48h-T) both CENP-A^Cnp1^ and CENP-C^Cnp3^ were detected on *cc2* (Fig. 2B). Thus, *cc2* borne on a linear minichromosome can be converted from a pre-chromatinised state to a CENP-A^Cnp1^ state. Moreover, colony colour assays indicate that hi-CENP-A^Cnp1^ expression induces increased loss of Ch16-*cc2*, which is consistent with a second functional kinetochore being formed at cc2 on Ch16 (Fig. 2C). Thus, Ch16-*cc2* behaves as an inducible dicentric chromosome controlled by CENP-A^Cnp1^ levels.

It is possible that high levels of CENP-A^Cnp1^ are continuously required to maintain CENP-A^Cnp1^ on pMcc2, or alternatively, once established, CENP-A^Cnp1^ and kinetochore proteins may persist even when CENP-A^Cnp1^ is returned to wild-type levels (wt-CENP-A^Cnp1^). To investigate the maintenance of CENP-A^Cnp1^ chromatin, pMcc2 was first transformed into hi-CENP-A^Cnp1^ cells to allow the assembly of centromeric chromatin and subsequently these pMcc2-containing cells were crossed with wt-CENP-A^Cnp1^ cells to transfer the pMcc2 plasmid into cells expressing wild-type CENP-A^Cnp1^ levels. ChIP analyses show that CENP-A^Cnp1^ persisted on the pMcc2 in this wild-type background (Fig. 2D). Western analysis of extracts from Parental, F1 and F2 cells confirmed that GFP-CENP-A^Cnp1^ was lost in F1 and F2 cells (Fig. 2D). Thus, CENP-A^Cnp1^ chromatin behaves as a true epigenetic entity in that once established it carries its own efficient propagation mechanism, persisting even though the original stimulus has been removed. More remarkably, this CENP-A^Cnp1^ chromatin is maintained through 2 rounds of meiosis and at least 50 mitotic divisions. Thus central domain sequences are particularly receptive to the establishment and maintenance of CENP-A^Cnp1^ chromatin.

### Central domain sequence and length affect *de novo* CENP-A^Cnp1^ deposition

**Fig 3:**
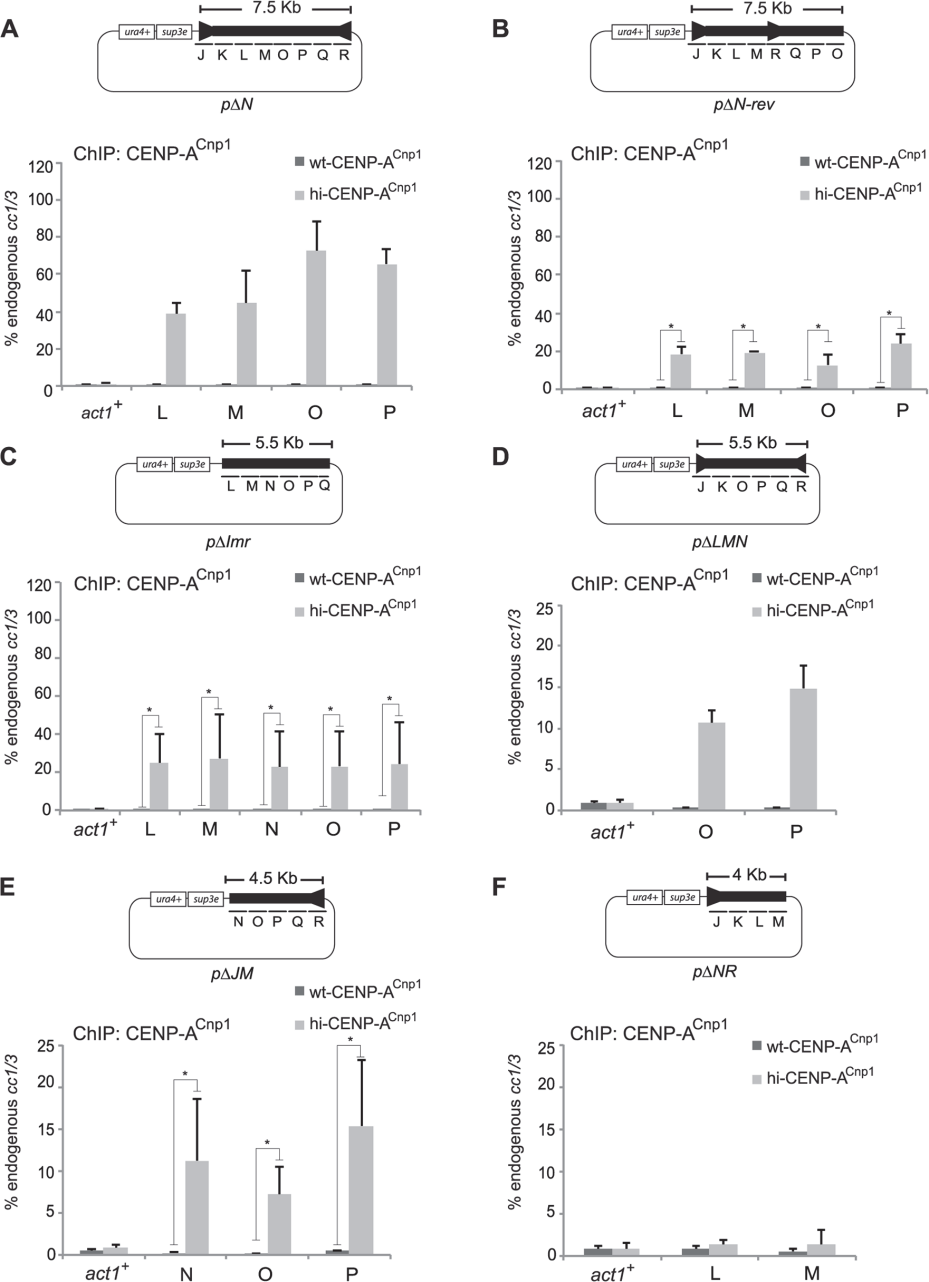
Centromeric DNA sequence affects CENP-A^Cnp1^ chromatin establishment. Plasmids containing fragments of cc2 DNA were transformed into wild-type (wt-CENP-A^Cnp1^) or cells expressing high levels of CENP-A^Cnp1^ (hi-CENP-A^Cnp1^). Plasmids contained: (A) Deletion of central 1 kb (N) from *cc2*, (B) Left half of *cc2*, (J to M: 4 kb) plus region O to R (3.5 kb) in inverted orientation (region N absent), (C) *imr2* regions (J and R) deleted along with 1 kb of *cc2* (K), leaving LMNOPQ, (D) Region L to N deleted, leaving JKOPQR. (E) Deletion of J to M, leaving N to R, (F) Deletion from N to R, leaving J to M. The enrichment of CENP-A^Cnp1^ on the plasmids was analysed by ChIP and calculated relative to the endogenous *cc1*/3 (n = 3). In (B), (C) and (E) *p-value* was calculated. Asterisks indicate *p* <0.05.

To determine if specific regions from the central domain of *cen2* are required to establish CENP-A^Cnp1^ chromatin, plasmids bearing different sub-fragments from *cc2* were transformed into wt-CENP-A^Cnp1^ or hi-CENP-A^Cnp1^ cells (Fig. 3). We used an unbiased approach to divide the 8.5 kb cc2 into 1 kb regions (J-R). Deletion of 1 kb from the centre of *cc2* (N) does not affect CENP-A^Cnp1^ establishment (pΔN; Fig. 3A, compare with pMcc2, Fig. 1B). Notably, CENP-A^Cnp1^ incorporation on a plasmid carrying identical centromeric DNA as pΔN but with the right half (O-R) inverted relative to the native sequence, was less efficient (pΔN-rev; Fig. 3B). Thus, the relative orientation of central domain sequences within *cc2* influences the degree of CENP-A^Cnp1^ deposition; directionality or the juxtaposition of certain sequences may be important for promoting CENP-A^Cnp1^ incorporation. The central CENP-A^Cnp1^ domain at endogenous fission yeast centromeres is composed of inverted *imr* repeats that flank the central core. ChIP analyses demonstrated that, in a plasmid-based establishment assay, the *imr* repeats are dispensable for *de novo* CENP-A^Cnp1^ incorporation on the remaining central domain sequences (pΔimr; Fig. 3C).

Deletion of additional regions (LMN) of *cc2* markedly decreased the efficiency of CENP-A^Cnp1^ incorporation relative to pMcc2 and pΔN **(** Fig. 3D; compare Fig. 1B, Fig. 3A), suggesting that either the LM region is critical for promoting CENP-A^Cnp1^ incorporation or that the overall reduced centromeric DNA length diminishes CENP-A^Cnp1^ deposition (Fig. 3D). However, further investigation using plasmids bearing smaller *cc2* fragments suggests that the specific sequences present have a more significant influence on CENP-A^Cnp1^ deposition than the overall length of *cc2* DNA present (Fig. 3E,F). For example, pΔJM and pΔNR differ by only 500 bp, however, pΔJM incorporated substantially more CENP-A^Cnp1^ than pΔNR (Fig. 3F). We conclude that specific sequences from the central domain of fission yeast centromeres, combined with their overall length, promote the efficient *de novo* assembly of CENP-A^Cnp1^ chromatin.

### A 2 kb region of centromeric DNA is sufficient to direct *de novo* CENP-A^Cnp1^ chromatin assembly

**Fig 4:**
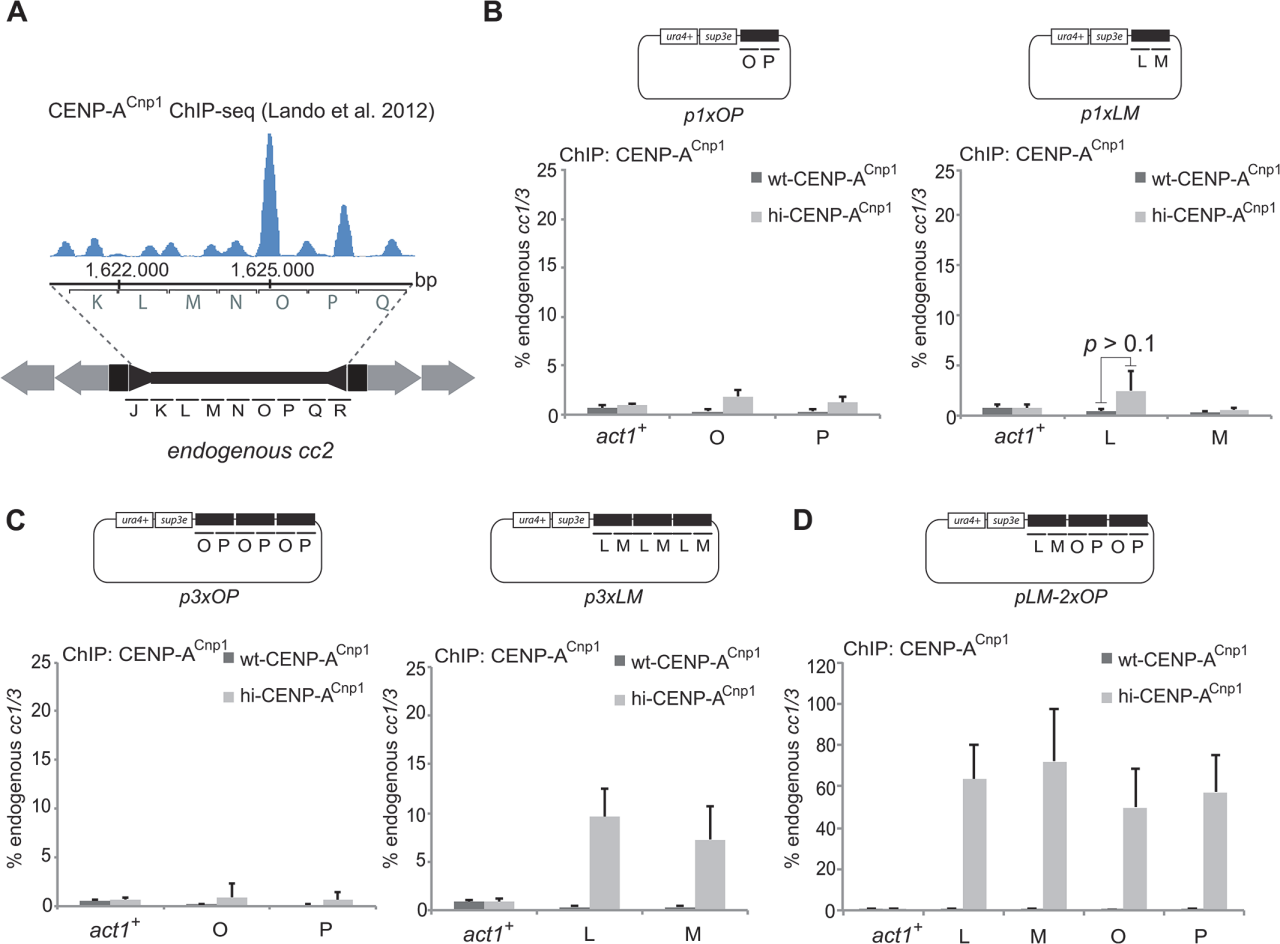
A 2 kb region of cc2 is sufficient to establish CENP-A^Cnp1^ chromatin. (A) Profile of ChIP-SEQ for CENP-A^Cnp1^ [49] and corresponding position on endogenous *cc2*. (B) ChIP analysis of CENP-A^Cnp1^ levels on plasmids containing one copy (p1xLM) or one copy of OP (p1xOP) transformed into wt-CENP-A^Cnp1^ or hi-CENP-A^Cnp1^. (C) CENP-A^Cnp1^ levels on plasmids containing three copies of a 2 kb fragment, either OP or LM, cloned in tandem repeats (p3xOP and p3xLM) and transformed into wild-type (wt-CENP-A^Cnp1^) or into cells expressing high levels of CENP-A^Cnp1^ (hi-CENP-A^Cnp1^). (D) A plasmid containing one copy of the LM fragment adjacent to two copies of OP (direct repeats) was transformed into wt-CENP-A^Cnp1^ and hi-CENP-A^Cnp1^ and CENP-A^Cnp1^ levels analysed by ChIP (n = 3).

It is possible that shorter fragments of centromere DNA from within the central domain can actively promote CENP-A^Cnp1^ assembly but that because longer total lengths are required to stabilise incorporated CENP-A^Cnp1^ the activity of shorter fragments cannot be detected. To address this possibility we selected two distinct sequences from the central domain of *cen2* for analyses. The 2 kb OP region was present on all the pMcc2 derivatives with which we detected significant CENP-A^Cnp1^ incorporation following transformation into hi-CENP-A^Cnp1^ cells (Fig. 3). In addition, ChIP-seq analysis indicates particularly high CENP-A^Cnp1^ nucleosome occupancy within OP at endogenous *cen2* [49 and Fig. 4A]. In contrast, the 2 kb LM region appears to be dispensable for *de novo* CENP-A^Cnp1^ assembly on pMcc2 derived plasmids and exhibits low CENP-A^Cnp1^ nucleosome occupancy (Fig. 3, Fig. 4A).

Initial tests showed that neither OP (p1xOP) nor LM (p1xLM) sequences alone were capable of inducing significant *de novo* CENP-A^Cnp1^ incorporation when introduced into hi-CENP-A^Cnp1^ cells (Fig. 4B). This finding is consistent with a minimal length of central domain DNA being required for stable CENP-A^Cnp1^ chromatin assembly and retention. To satisfy this apparent length requirement, the OP and the LM fragments were multimerised as tandem repeats to create 3xOP and 3xLM (p3xOP, p3xLM; Fig. 4C). Remarkably, when transformed into hi-CENP-A^Cnp1^ cells no CENP-A^Cnp1^ was detectable on p3xOP whereas p3xLM allowed a reasonable level of CENP-A^Cnp1^ incorporation (Fig. 4C). This suggests that in isolation the OP region is unable to promote CENP-A^Cnp1^ deposition even though in the context of an entire central domain it normally accepts CENP-A^Cnp1^ and ends up with high CENP-A^Cnp1^ nucleosome occupancy (Fig. 4A). We note that the removal of the LM region from the central domain of pMcc2 derived plasmids greatly reduced the level of CENP-A^Cnp1^ incorporated on OP (compare pΔN Fig. 3A with pΔLMN Fig. 3D). Thus, in contrast to OP, the LM region appears to have the ability to induce CENP-A^Cnp1^ deposition. To directly test this possibility, a single copy of LM was placed adjacent to two tandem copies of OP (pLM-2xOP) and transformed into hi-CENP-A^Cnp1^ cells. High levels of CENP-A^Cnp1^ were detected on both the LM and OP regions of pLM-2xOP (Fig. 4D), thus the LM region has an innate ability to stimulate CENP-A^Cnp1^ deposition on the OP region. A different arrangement of the same sequences (pOPLMOP) also attracted CENP-A^Cnp1^ in hi-CENP-A^Cnp1^ cells (S2 Fig.). These analyses indicate that the 2 kb LM sequence contains all the features that are required to promote and accept CENP-A^Cnp1^ assembly, and thus LM defines a 2 kb region of *S*. *pombe* centromeric sequence that allows the *de novo* assembly of CENP-A^Cnp1^ chromatin.

### The 2 kb LM element is sufficient to form functional centromeres

**Fig 5:**
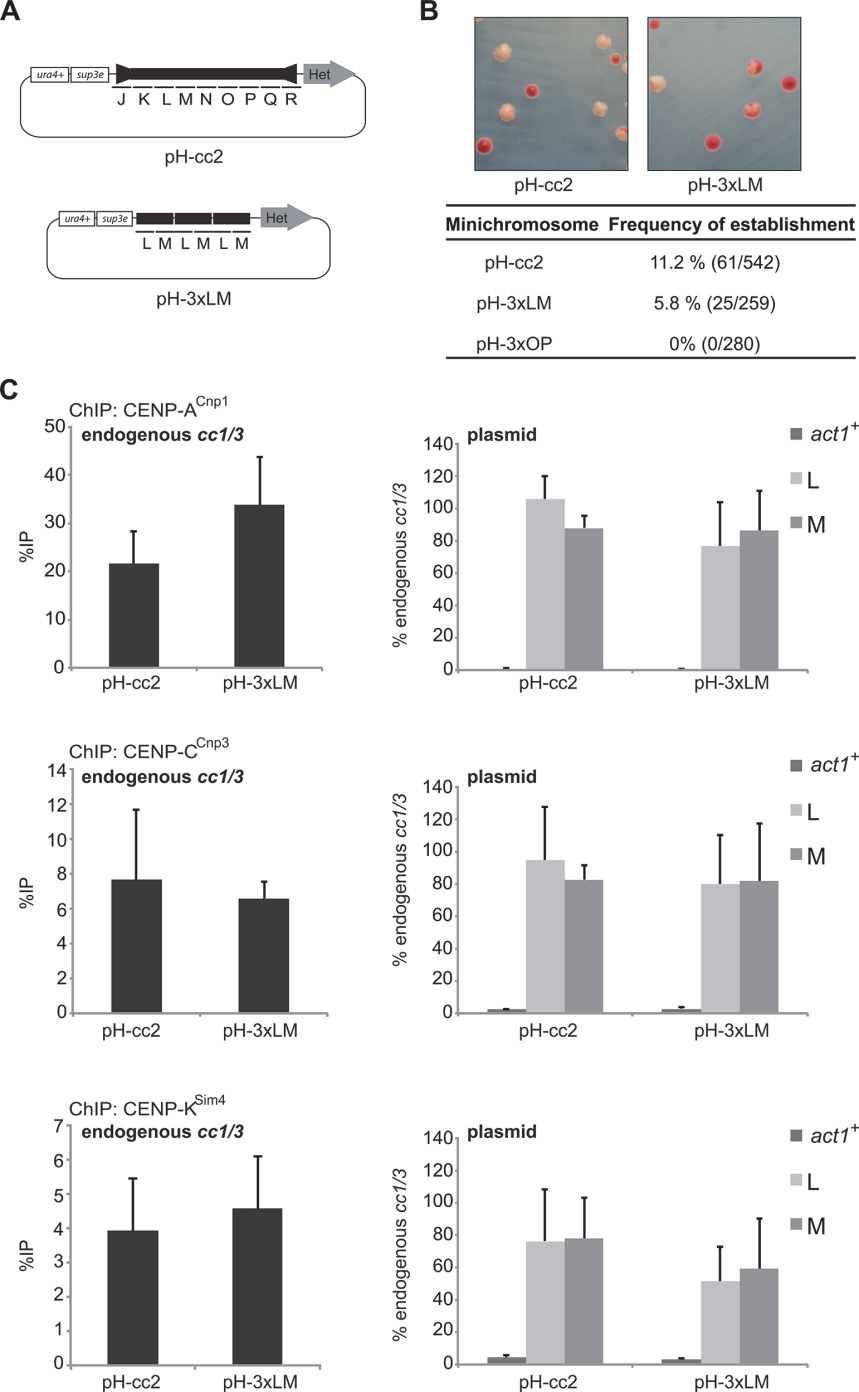
Tandem copies of the 2 kb LM region adjacent to heterochromatin/outer repeat are sufficient to establish a functional centromere in wild-type cells. (A) Schematic representation of plasmids containing 5.6 kb of heterochromatin-forming outer repeat element flanking a full length *cc2* (pH-cc2) or three tandem repeats of the LM region (pH-3xLM). (B) Establishment assay in wild-type cells. pH-cc2, pH-3xLM and pH-3xOP were transformed in wild-type cells and the percentage of transformants that contained plasmids with centromere function was assessed. White colonies with red sectors indicate formation of functional centromeres (details in M&M). (C) ChIP analysis of CENP-A^Cnp1^, CENP-C^Cnp3^ and CENP-K^Sim4^ levels reported as %IP for endogenous centromeres (*cc1/3*) and relative to *cc1/3* at *act1*+ and on plasmids (L and M) (n = 3).

Plasmids bearing an entire central core domain flanked by outer heterochromatin repeats assemble functional centromeres when transformed into wild-type cells [45,50]. To determine if the 2 kb LM region imparts centromere function, a plasmid carrying the 3xLM tandem repeat adjacent to a 5 kb outer repeat heterochromatin forming element (pH-3xLM) was transformed into wild-type cells expressing CENP-A^Cnp1^ at normal levels (Fig. 5A). The establishment of functional centromeres in the resulting transformants was monitored by an *ade6*-based colony colour sectoring assay [51]. Minichromosomes carrying full-length *cc2* and 5 kb of outer repeat heterochromatin were able to establish functional centromeres upon transformation (Fig. 5B and S2 Fig.). pH-3xLM and pH-LM-2xOP transformants also established functional centromeres, but at lower frequency than pH-cc2 (Fig. 5B and S2 Fig.). Differences in the ability of various constructs to form functional centromeres may reflect the particular configuration of sequences in individual minichromosomes. In contrast, pH-3xOP (3xOP flanked by heterochromatin) was unable to establish functional centromeres. Thus the LM sequence in a 3x tandem array, flanked by heterochromatin, is sufficient to form functional centromeres. ChIP analyses confirmed that kinetochores were assembled on pH-3xLM since CENP-A^Cnp1^ and the kinetochore proteins CENP-C^Cnp3^ and CENP-K^Sim4^ were enriched over the LM sequences at levels comparable to endogenous centromeres (Fig. 5C). We conclude that the LM sequence within pH-3xLM not only promotes incorporation of CENP-A^Cnp1^ into chromatin but also supports the assembly of a functional centromere.

### Features within centromeric central domain DNA are required to promote CENP-A^Cnp1^ chromatin establishment

**Fig 6:**
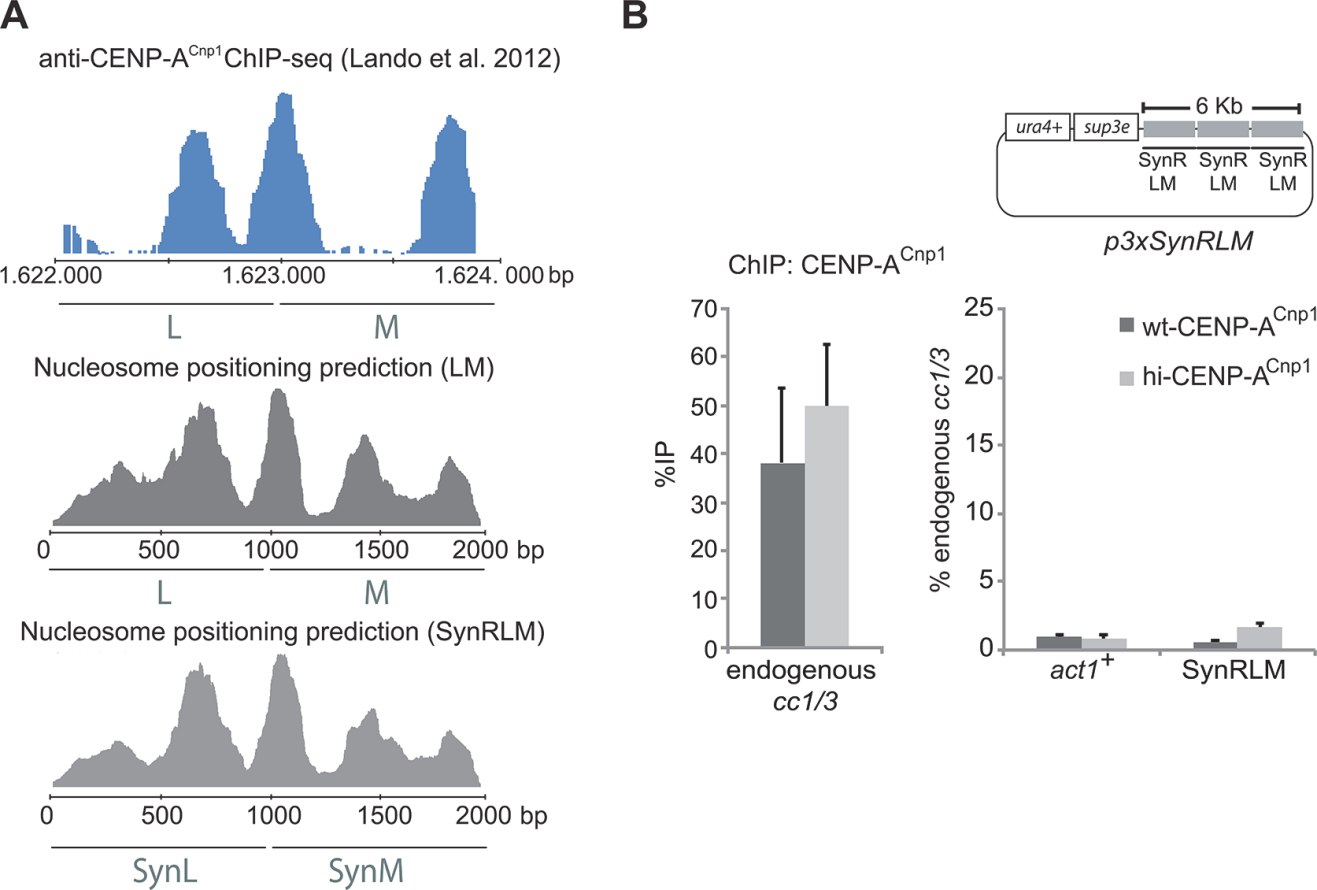
Genetic information contained within DNA sequence is required for centromeric chromatin establishment. (A) Comparison between ChIP-seq data for CENP-A^Cnp1^([49]; blue) and nucleosome positioning prediction (algorithm developed by [55]; in grey) for the LM region of *cc2* or a mutagenised synthetic version of LM (SynRLM). The algorithm predicts nucleosome peaks within the central domain that match those mapped *in vivo* by ChIP-seq. SynRLM was designed by randomising the LM sequence in a 5 bp window and taking into consideration both periodicity of nucleosome positions and AT/GC content distribution of the original LM sequence. (B) ChIP analysis of CENP-A^Cnp1^ levels of wild-type (wt-CENP-A^Cnp1^) and cells overexpressing CENP-A^Cnp1^ (hi-CENP-A^Cnp1^) transformed with a plasmid containing three tandem repeats of SynRLM (p3xSynRLM). CENP-A^Cnp1^ levels are shown relative to *cc1/3* (n = 3).

Nucleosome occupancy is known to be influenced by a combination of DNA sequence and the action of chromatin remodelers [52]. Primary DNA sequence itself influences nucleosome occupancy since DNA sequences with a high GC content and periodic dinucleotide patterns, that are devoid of poly(dA:dT) sequences, are strongly favored for nucleosome occupancy because of biophysical constraints that allow such sequences to wrap more easily around nucleosomes. These constraints have led to the development of algorithms that predict the probability of nucleosome occupancy [53,54]. In common with centromeres of many organisms, fission yeast centromeric DNA is AT-rich with a higher frequency of poly(dA:dT) tracts. It is therefore possible that H3 nucleosomes have a lower affinity for such sequences whereas CENP-A nucleosomes may be unperturbed by such AT rich DNA. To examine the underlying sequence specificity within centromeric DNA that favours the deposition of CENP-A^Cnp1^ nucleosomes, the sequence of LM DNA was altered by randomisation using a 5 bp sliding window throughout the entire 2 kb element. This generated a synthetic LM sequence (SynR-LM) that is 62.6% identical to the wild-type LM sequence, retaining the same AT content and dinucleotide periodicity, and thus the same predicted nucleosome occupancy as the wild-type LM element (Fig. 6A) [55].

Synthesised SynR-LM assembled as a 3xSynR-LM tandem array was placed in the same plasmid backbone as p3xLM to generate p3xSynR-LM. p3xSynR-LM was transformed into wt-CENP-A^Cnp1^ and hi-CENP-A^Cnp1^ cells. In contrast to p3xLM, CENP-A^Cnp1^ was not detectable on the shuffled LM sequence of pSynR-LM (Fig. 6B, compare with Fig. 4B). These analyses demonstrate that preservation of nucleotide composition (AT-content, dinucleotide periodicity) and predicted nucleosome occupancy within an altered centromeric DNA is not sufficient to allow CENP-A^Cnp1^ deposition. The fact that the natural 2 kb LM sequence is active whereas the artificial SynR-LM is inactive reveals that the primary sequence of wild-type centromeric LM DNA encodes properties that somehow allow its recognition *in vivo* and consequent *de novo* assembly of CENP-A^Cnp1^ chromatin.

### Centromeric DNA produces an unusual transcriptional environment

**Fig 7:**
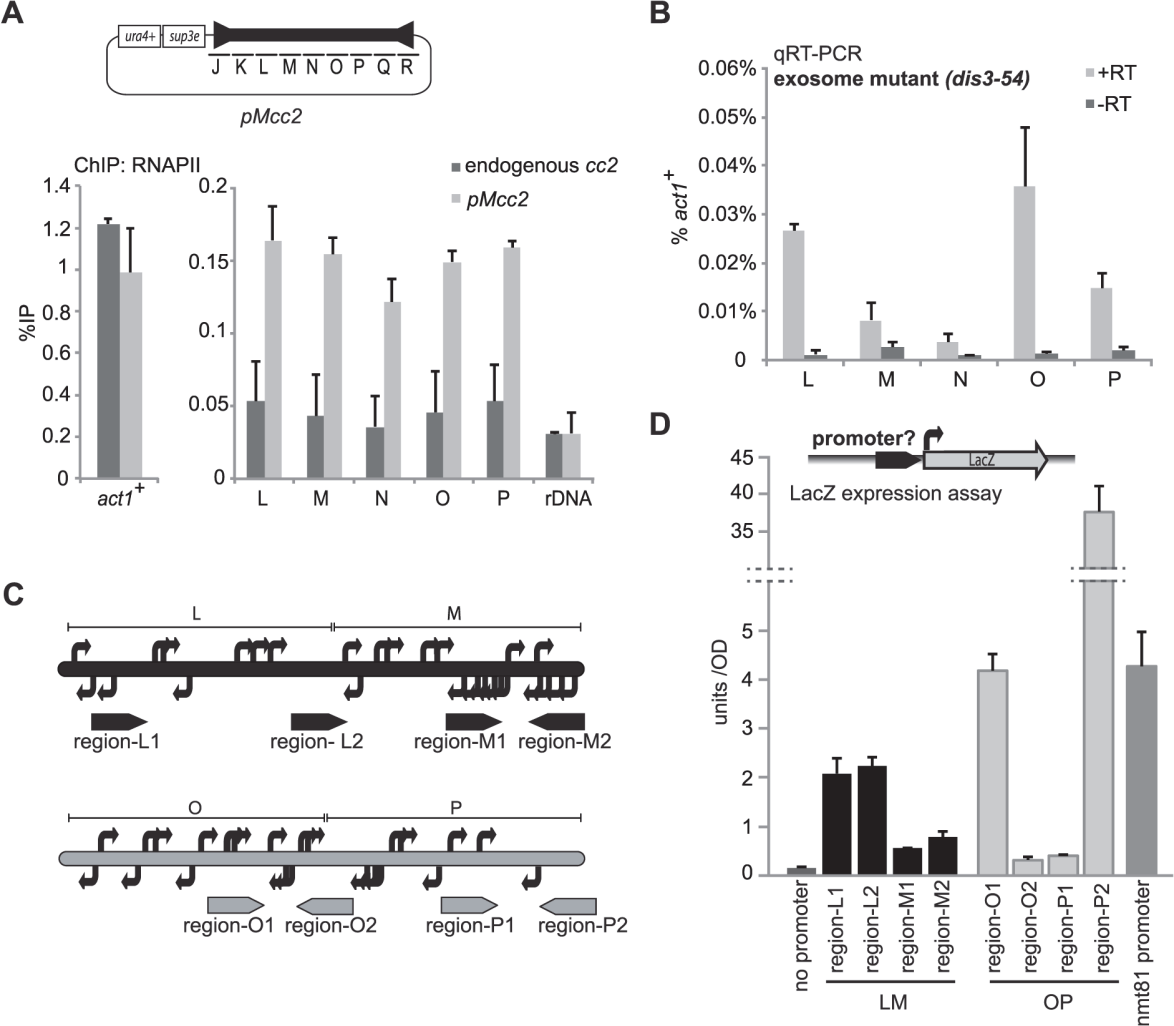
High levels of RNAPII but low levels of transcripts are found at central domain. (A) ChIP of RNA polymerase II (RNAPII) at endogenous *cc2* and at the pMcc2 plasmid in wild-type cells (n = 3). rDNA: negative control. (B) qRT-PCR performed on total RNA extracted from exosome mutant cells (*dis3–54*) lacking endogenous *cc2* and transformed with the pMcc2 plasmid (n = 3). Transcript levels are shown relative to *act1* ^+^ (n = 3). (C) 5’RACE-PCR was performed on poly(A) purified RNA extracted from exosome mutant (*dis3–54*) cells lacking endogenous *cc2* and transformed with the p3xLM or p3xOP plasmid. In the figure, TSSs are represented as bent arrows. (D) Analysis of promoter activity: ∼200 bp fragments from *cc2* (region-L1,-L2,-M1,-M2,-O1,-O2,-P1,-P2) were placed upstream of a LacZ reporter gene. The levels of LacZ expression was assessed by measuring absorbance at 420 nm of cell lysates incubated with 2-Nitrophenyl-β-D-galactopyranoside (ONPG). nmt81: positive control with *nmt81* promoter (n = 3).

Upon transformation into cells innate features within 3xLM sequence must allow it to be either immediately assembled in CENP-A^Cnp1^ chromatin, or, initially assembled in H3 chromatin with subsequent remodelling that exchanges canonical H3 for CENP-A^Cnp1^. The process of transcription is obviously accompanied by chromatin remodelling and non-coding transcripts synthesised from within the central CENP-A^Cnp1^ domains of fission yeast centromeres have been detected [37,42]. The transcription of central domain DNA might influence the assembly of CENP-A^Cnp1^ chromatin. In cells expressing CENP-A^Cnp1^ at wild-type levels, plasmid-borne central domain sequences are assembled in H3 rather than CENP-A^Cnp1^ chromatin (Fig. 1E). Higher levels of RNAPII are detected on plasmid-borne central domain sequences (pMcc2) introduced into wild-type cells than when *cc2* is assembled in CENP-A^Cnp1^ chromatin on pMcc2 or at endogenous centromeres (Fig. 7A, S3 Fig.). Although relatively high levels of RNAPII associate with the pMcc2 central domain when assembled as H3 chromatin in wild-type cells (10–30% of levels at *act1* ^*+*^) (Fig. 1E, Fig. 7A), the level of transcripts emanating from the central domain is very low (<0.1% of *act1* ^*+*^), even when analysed in exosome defective cells (*dis3–54*; Fig. 7B). Thus, although ample RNAPII is recruited to the central domain of pMcc2 few transcripts are generated, suggesting that transcriptional stalling occurs.

To map transcriptional start sites (TSSs) within the LM and OP regions, 5’ RACE was performed on RNA extracted from *dis3–54* exosome mutant cells harbouring p3xLM or p3xOP (Fig. 7C, S4 Fig.). Many TSSs were identified within LM and OP, suggesting that these regions contain several promoters (Fig. 7C). 200 bp regions from both LM and OP were tested for their ability to drive production of β-galactosidase when placed upstream of a *lacZ* reporter in fission yeast and as shown in Fig. 7D, the regions displayed promoter activity. Mutated or inverted versions of promoter region M2 did not promote transcription of LacZ (S4 Fig.). Whilst most regions of LM and OP exhibit promoter activity that is lower than that of *nmt81* control promoter, it is notable that region-O1 and region-P2 from OP have equivalent and 10-fold higher activity, respectively (Fig. 7D). It is possible that the higher promoter activity possessed by some regions of OP may affect its ability to establish CENP-A^Cnp1^. We surmise that the central domain from *cen2* is peppered with promoters that can drive the production of transcripts on both strands. Their relative arrangement along with the strength and pattern of transcription may affect CENP-A^Cnp1^ incorporation.

### CENP-A^Cnp1^ establishment is enhanced in mutants that increase RNAPII stalling

The progression of RNAPII is impeded by obstacles such as nucleosomes, DNA damage, bound proteins and by sequences that are intrinsically difficult to transcribe, causing transcriptional pausing, stalling or arrest [56]. RNAPII-associated proteins ease the passage of RNAPII through such impediments, contributing to the processivity of the polymerase [57]. TFIIS facilitates transcriptional elongation of stalled/backtracked RNAPII by stimulating cleavage of nascent transcripts [58–60]. Upon stalling an elongating RNAPII becomes mono- then poly-ubiquitylated on the largest Rpb1 subunit. A rescue pathway involving de-ubiquitylation by the ubiquitin hydrolase Ubp3 is deployed to restart stalled RNAPII [56,61].

**Fig 8:**
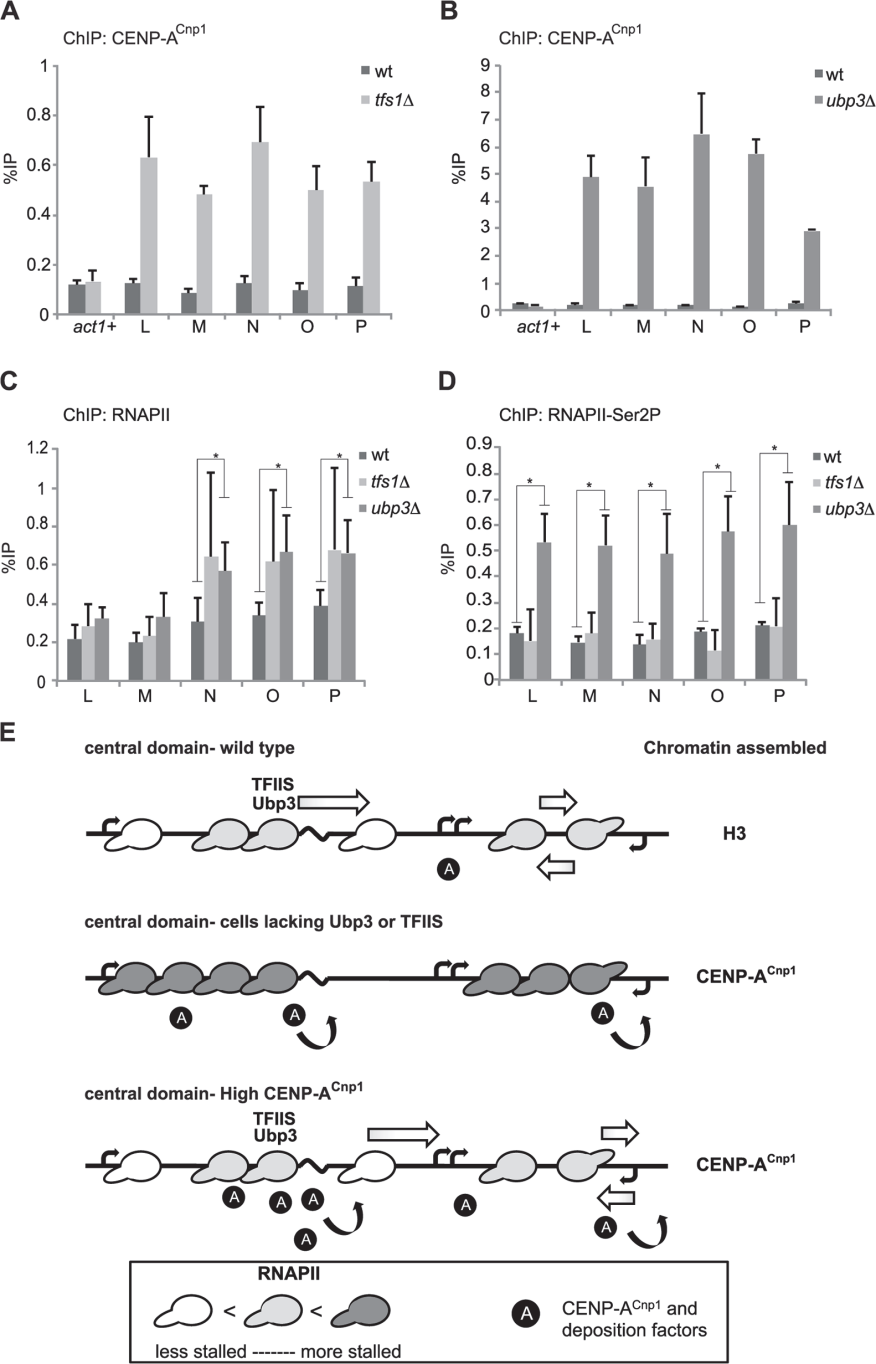
Mutants that affect RNAPII elongation allow *de novo* establishment of CENP-A^Cnp1^ chromatin. (A) ChIP for CENP-A^Cnp1^ on a pMcc2 plasmid transformed in wild-type (wt) or a strain deleted for TFIIS^Tfs1^ (*tfs1Δ*) (n = 3). (B) ChIP for CENP-A^Cnp1^ on a pMcc2 plasmid transformed in wild-type (wt) or a strain deleted for Ubp3 (*ubp3*Δ) (n = 3). (C) ChIP for RNAPII on pMcc2 plasmid in wt, *tfs1Δ* and *ubp3Δ*. *p<0*.*05* is indicated with an asterisk. (D) Enrichment of RNAPII phosphorylated at Ser2 of its CTD (RNAPII-Ser2P) at the pMcc2 plasmid in wt, *tfs1*Δ and *ubp3*Δ. *p<0*.*05* is indicated with an asterisk (n = 3) (E) Model for establishment of CENP-A^Cnp1^ on central domain DNA. *Top panel*: Central domain DNA contains numerous weak promoters on both strands, leading to collisions between RNAPII complexes and stalling (depicted on right side). In addition, regions that are difficult to transcribe (wavy black line) may cause frequent stalling of RNAPII. RNAPII is depicted as an elongated ellipse and depth of shading indicates degree of stalling. Stalling is transient as factors such as TFIIS and Ubp3 help clear stalling so that elongation of RNAPII can proceed. Shaded-white arrow represents restarting of transcription. *Middle panel*: Persistent stalling occurs in cells lacking Ubp3 or TFIIS and this RNAPII status attracts deposition factors leading to the incorporation of CENP-A^Cnp1^ in central domain chromatin. Black circles labelled ‘A’ represent CENP-A^Cnp1^ and deposition factors. Curved black arrows represent CENP-A^Cnp1^ deposition. *Bottom panel*: Even transient RNAPII stalling is sufficient to recruit CENP-A^Cnp1^ and deposition factors when CENP-A^Cnp1^ is present at high levels.

Our analyses suggest that the central domain chromatin landscape contains numerous promoters on both strands and multiple TSSs. In addition, long poly(dA:dT) tracts are likely to be an intrinsically problematic sequence for RNAPII transcription and present a barrier to RNAPII elongation [62,63]. We reasoned that mutants that are defective in the response to transcriptional stalling might influence the ability of the central domain to become assembled in CENP-A^Cnp1^ chromatin. To test this possibility, wild-type and TFIIS (*tfs1Δ*) mutant cells expressing hi-CENP-A^Cnp1^ were transformed with pMcc2. Surprisingly, slightly increased levels of CENP-A^Cnp1^ were detected on pMcc2 in the *tfs1Δ* mutant compared to wild-type cells, suggesting that loss of TFSII promotes CENP-A^Cnp1^ deposition (S5 Fig.). Consistent with this, even when pMcc2 was transformed into *tfs1Δ* cells expressing wt-CENP-A^Cnp1^ levels, CENP-A^Cnp1^ was detected on the pMcc2 central domain (Fig. 8A). In order to determine whether the effect on CENP-A^Cnp1^ establishment was specific to *tfs1Δ* or a general consequence of increased RNAPII stalling, we also investigated if loss of the ubiquitin hydrolase Ubp3, which normally rescues arrested RNAPII, affects CENP-A^Cnp1^ deposition. Strikingly, CENP-A^Cnp1^ was detected at high levels on central domain sequences in *ubp3Δ* cells transformed with pMcc2. CENP-A^Cnp1^ was also detected on p3xLM, but not p3xOP in *ubp3*Δ (Fig. 8B, S6 Fig.). CENP-C^Cnp3^ and CENP-K^Sim4^ centromere proteins were also significantly enriched on pMcc2 in *ubp3Δ* cells (S8 Fig.). These effects were not due to increased abundance of CENP-A^Cnp1^ in *tfs1Δ* or *ubp3Δ* cells as protein levels were similar to wild-type cells (S7 Fig.). In fact, a reduction in CENP-A^Cnp1^ and CENP-C^Cnp3^ levels was detected at endogenous centromeres in *ubp3*Δ, but not *tfs1*Δ cells (S8 Fig.). Tfs1 and Ubp3 were previously reported to modulate RNAi-independent heterochromatin assembly [64]. To test whether the effect on CENP-A^Cnp1^ establishment in *tfs1Δ* or *ubp3Δ* cells could be due to spurious assembly of heterochromatin on pMcc2, H3K9me2 ChIP was performed. The level of H3K9me2 on pMcc2 in *tfs1Δ*and *ubp3Δ* was similar to that on a negative control locus, *act1* ^*+*^, and assembly of CENP-A^Cnp1^ on pMcc2 in these mutants was not dependent on the H3K9-methyltransferase Clr4 (S9 Fig.). Thus, CENP-A^Cnp1^ assembly on pMcc2 in the absence of TFIIS or Ubp3 does not result from induction by ectopic heterochromatin.

If lack of TFIIS or Ubp3 hinders transcriptional elongation, an increased level of RNAPII would be expected on affected chromatin templates. Elevated levels of Rpb1/RNAPII were detected on the central domain of pMcc2 in *tfs1Δ* (TFIIS) and *ubp3Δ* cells (Fig. 8C). In addition, increased levels of the elongation-specific Phospho-Ser2 form of RNAPII were observed on the central domain of pMcc2 in *ubp3Δ* cells, suggestive of failure to efficiently clear stalled RNAPII (Fig. 8D). Thus, two mutants, which perturb the progress of RNAPII elongation complexes in different ways, lead to deposition of CENP-A^Cnp1^. These observations suggest that altering the transcriptional properties of the central domain chromatin through increased RNAPII stalling creates an environment that is permissive for establishment of CENP-A^Cnp1^ chromatin in place of H3 chromatin.

## Discussion

It is thought that once established, CENP-A chromatin has the ability to be ‘self-propagating’, and through the recruitment of factors that are themselves involved in deposition of CENP-A, it ensures its own maintenance [16,17,23,24,26,65]. Epigenetic inheritance can be defined as the propagation of a state in the absence of the initial inducer of that state. In this study, the inducer—overexpression of CENP-A^Cnp1^—causes an event that would not normally occur, the assembly of CENP-A^Cnp1^ chromatin on episomal centromeric DNA (pMcc2). When CENP-A^Cnp1^-assembled pMcc2 is crossed from hi-CENP-A^Cnp1^ cells into wt-CENP-A^Cnp1^ cells, CENP-A^Cnp1^ is propagated in the absence of the initial inducer through many generations and through meiosis. These observations further strengthen the evidence that CENP-A behaves as a bona fide epigenetic entity [24].

It is clear that both epigenetic and genetic factors influence CENP-A assembly. We have investigated the role of DNA sequence in establishment of CENP-A chromatin in fission yeast, an organism where analysis is not confounded by repetitive arrays of short satellite sequences. CENP-A^Cnp1^ is normally restricted to the central domain of centromeres where it forms the basis for the kinetochore. Central domain DNA is a preferred substrate for establishment of CENP-A^Cnp1^ chromatin upon overexpression, whilst other genomic loci do not support accumulation of high levels of CENP-A^Cnp1^ [47], and even vector DNA adjacent to the central domain is not a good substrate. Conditions and mechanisms that influence assembly of CENP-A^Cnp1^ on naïve plasmid DNA are also able to convert pre-chromatinised *cc2* present on episomal plasmids or linear minichromosomes. What makes central domain DNA a preferred site for CENP-A^Cnp1^ assembly? The lack of homology between *cc2* and *cc1/cc3* sequences suggests that it is not a simple case of specific sequence that is critical [66–68]. Our analyses indicate that there is functional redundancy within the central domain and no one particular sequence is either necessary or sufficient for CENP-A^Cnp1^ establishment, consistent with previous findings [50]. Despite this redundancy it appears that there are inherent distinctions between different regions of *cc2*. The 2 kb sub-regions, LM and OP, are functionally non-equivalent and consistently behaved differently when challenged to assemble CENP-A^Cnp1^ chromatin. LM is competent to establish centromeric chromatin upon CENP-A^Cnp1^ overexpression, contains sufficient information to make a functional centromere when placed next to heterochromatin (pH-3xLM), and assembles CENP-A^Cnp1^ chromatin in cells lacking Ubp3. On the other hand, the OP region fails to become assembled in CENP-A^Cnp1^ chromatin in all these situations, yet can accept CENP-A^Cnp1^ when adjacent to one copy of LM, which apparently acts as an initiator. The ability of LM, but not OP, to substitute for full-length *cc2* sequence indicates that not all sequences are equivalent and LM must contain all information necessary to make this region permissive for CENP-A^Cnp1^ establishment. It is possible that the observed higher promoter activity observed in the OP region (Fig. 7D) prevents stabilisation of CENP-A^Cnp1^ nucleosomes on this sequence.

In common with many organisms, the central domain of *S*. *pombe* centromeres is AT rich and this property might contribute to the propensity of centromeric DNA to attract CENP-A [5,68]. *S*. *pombe* central domain DNA has an AT content of 72% (genome average of 64%), as does the establishment competent LM sequence. However, other regions that alone fail to support CENP-A^Cnp1^ establishment have a similar AT content, such as OP (71% AT) and intergenic regions (72% AT). Moreover, randomisation of the LM sequence resulted in SynR-LM that, even with identical nucleotide composition (72% AT), was incompetent for CENP-A^Cnp1^ establishment. Thus, high AT content alone, even when it mimics natural nucleosome positioning predictions, is not a defining factor in CENP-A^Cnp1^ assembly. Together our observations indicate that rather than there being a specific critical sequence, central domain sequences encode unique properties capable of triggering or promoting the establishment of CENP-A^Cnp1^ chromatin.

Transcription-coupled remodelling is associated with the deposition of histone variants and could potentially contribute to the assembly of CENP-A chromatin [69,70]. However, the simple act of transcription cannot be sufficient to provide specificity to the deposition of CENP-A. Our observations suggest that the transcriptional landscape of the centromeric central domain is unusual: scattered promoters of various strengths resulting in pervasive low quality transcription and numerous TSSs on both strands, in conjunction with poly(dA:dT) tracts that are inherently difficult to transcribe are likely to cause collision between convergently transcribing RNAPIIs and pile-ups at difficult sequences [63,71]. The relatively high density of RNAPII on pMcc2 contrasts with very low levels of transcripts (Fig. 7), consistent with inefficient progress of transcription by RNAPII on *cc2*, and many stalled elongation complexes. In addition, long tracts of poly(dA:dT) are known to disfavour nucleosome assembly, consistent with the apparently wide spacing of nucleosomes at endogenous centromeres [49,72]. These regions may be *de facto* nucleosome free regions, similar to those at promoters, allowing cryptic initiation of transcription to occur [72,73]. The randomized synthetic sequence SynR-LM that is a poor substrate for CENP-A^Cnp1^ deposition has similar long A tracts, but transcription-related sequence-sensitive elements—such as promoters and transcription factor binding sites—would be destroyed. Thus, the central domain, due to its sequence-encoded properties, may produce a distinctive chromatin and transcriptional environment.

CENP-A^Cnp1^ chromatin does not assemble *de novo* on *cc2* sequence alone in wild-type cells expressing normal CENP-A^Cnp1^ levels [45]. Instead, we envisage that the unique transcriptional chromatin environment created by the *cc2* sequence renders it permissive for CENP-A^Cnp1^ establishment, but that establishment occurs only if other favourable conditions exist. CENP-A^Cnp1^ is preferentially incorporated on these central domain sequences upon overexpression, when adjacent to heterochromatin, and in the absence of factors that usually enhance transcriptional elongation. Any explanation of CENP-A^Cnp1^ chromatin establishment on central domain DNA must also account for how CENP-A^Cnp1^ is incorporated instead of H3. Serine 2 in the CTD heptad repeat of Rpb1 is phosphorylated in elongating RNAPII, and this Ser2P-Rbp1/RNAPII becomes ubiquitylated upon stalling [74–76]. The ubiquitin hydrolase Ubp3 normally acts as a proof-reading activity to prevent degradation of stalled but rescuable RNAPII [56,61]. Absence of Ubp3 compromises the processing of stalled RNAPII, resulting in the accumulation of ubiquitylated Ser2P-Rbp1/RNAPII complexes. We propose that such modifications contribute to the distinctive status of central domain chromatin, leading to recruitment of factors that promote CENP-A^Cnp1^ deposition (Fig. 8E). Alternatively, it may create an environment in which H3 nucleosomes are efficiently turned over/evicted, whereas CENP-A^Cnp1^ nucleosomes are poorly evicted specifically in the context of stalled RNAPII. In cells lacking Ubp3, severe or prolonged stalling, even with normal levels of CENP-A^Cnp1^, would provide extended opportunities for CENP-A^Cnp1^ recruitment, or poor eviction of CENP-A^Cnp1^ during prolonged stalling. TFIIS promotes transcriptional elongation by cleaving nascent transcripts in the context of stalled/backtracked RNAPII [57,58,77]. Although the effects of TFIIS deletion are more subtle than lack of Ubp3, the accumulation of RNAPII correlates with assembly CENP-A^Cnp1^ chromatin, supporting a mechanism where persistent RNAPII stalling within central domain triggers remodelling that results in CENP-A^Cnp1^ deposition.

In this model, when naïve central domain DNA (pMcc2) is introduced into wild-type cells, transient stalling occurs but it is efficiently cleared with the aid of factors such as TFIIS and Ubp3 (Fig. 8E). Because in wild-type cells CENP-A^Cnp1^ levels are extremely low compared to histone H3 there would be little opportunity for CENP-A^Cnp1^ to gain access to *cc2*, and with efficient clearing of stalled RNAPII, CENP-A^Cnp1^ would fail to accumulate in *cc2* [78]. CENP-A^Cnp1^ overexpression would increase the probability of interaction with the transiently stalled RNAPII in central domain chromatin, increasing the likelihood of recruitment. Alternatively, increased access coupled with poor eviction would lead to CENP-A^Cnp1^ accumulation. In addition, CENP-A^Cnp1^ nucleosomes themselves, which have distinct N-terminal tails that lack the conserved lysine residues of H3 whose modification aids transcription, are likely to present a greater barrier to transcription than H3 nucleosomes [79]. Thus, once incorporated, CENP-A^Cnp1^ nucleosomes might exacerbate the poor transcriptional elongation, creating conditions permissive for recruitment of more CENP-A^Cnp1^ in a self-perpetuating system. Longer regions of central domain DNA would have greater probability of triggering stalling events and thus be more likely to initiate the incorporation of CENP-A^Cnp1^. In the context of this model, heterochromatin could promote establishment of CENP-A^Cnp1^ chromatin on adjacent *cc2* sequence by drawing plasmids to sites of endogenous heterochromatin such as the spindle pole body where they would encounter a higher concentration of CENP-A^Cnp1^ than non-heterochromatinized plasmids located in the nuclear interior [80]. Alternatively, heterochromatin-associated chromatin modifying activities may influence transcriptional elongation by RNAPII within *cc2*, causing enhanced stalling and deposition of CENP-A^Cnp1^[41].

Following establishment of CENP-A chromatin and kinetochore assembly, transcription could play a proof-reading role that evicts H3 deposited at centromeres during S phase [81]. Indeed, transcription and RNAPII have been detected at centromeres in mammalian cells and transcription/RNAPII may play a role in centromere integrity [33,34,38]. Transcription of human α-satellite arrays introduced as HACs is known to occur. Although CENP-A assembly is compatible with targeting of mild transcriptional activators, targeting of a strong transcriptional activator is deleterious [30,38,82]. Thus transcription and/or the transcription-coupled histone modifications detected at centromeres may promote CENP-A deposition at mammalian centromeres.

In conclusion, we show that the sequence of fission yeast centromere central domain DNA is important only in so far as it encodes for certain properties that contribute to the region’s unusual chromatin and transcriptional landscape. Establishment of CENP-A^Cnp1^ chromatin is driven by these sequence-encoded properties that when combined with the presence of nearby heterochromatin, overexpressed CENP-A^Cnp1^ or increased RNAPII stalling, tips the balance in favour CENP-A^Cnp1^ chromatin assembly. It seems likely that a similar combination of factors, which together favour CENP-A incorporation, must also contribute to the formation of neocentromeres at novel chromosomal locations.

## Materials and Methods

### Cell growth and manipulation

Standard genetic and molecular techniques were followed. Fission yeast methods were as described [83]. Fission yeast strains are listed in Table 1. Minichromosomes used in this study were transformed by electroporation. Transformants were selected by growth on PMG—ura—ade at 32°C. As circular minichromosomes lack heterochromatin and therefore centromeric cohesion, plasmids were maintained in cells by selection in medium lacking adenine and uracil. 3 independent colonies from each transformation were analysed for the presence of kinetochore proteins by chromatin immunoprecipitation (ChIP).

**Table 1:** *Schizosaccharomyces pombe* strains.

A7373	*cc2Δ*::*cc1 ade6-704-HYGMX6 ura4-*
A7408	*cc2Δ*::*cc1 ars1*:*nmt41-GFP-cnp1-NAT ade6-704-HYGMX6 ura4-*
A7528	*cc2Δ*::*cc1 dis3–54 ade6-704-HYGMX6 ura4-*
A9235	*cc2Δ*::*cc1 tfs1Δ*::*leu2 ade6-704-HYGMX6 ura4-*
A9391	*cc2Δ*::*cc1 ubp3Δ*::*KAN ade6-704-HYGMX6 ura4-*
A9491	*cc2Δ*::*cc1 tfs1Δ*::*leu2 clr4Δ*::*NAT ade6-704-HYGMX6 ura4-*
A9477	*cc2Δ*::*cc1 ubp3Δ*::*KAN clr4Δ*::*NAT ade6-704-HYGMX6 ura4-*
A9048	*cc2Δ*::*cc1 [Ch16 m23*:*ura4*::*cc2-KAN ade6-216] ade6-210 leu1-32 ura4-*
A9054	*cc2Δ*::*cc1 [Ch16 m23*:*ura4*::*cc2-KAN ade6-216] ars1*:*nmt41-GFP-Cnp1-NAT ade6–210 ura4-*
A9059	*cc2Δ*::*cc1 [Ch16-ade6-216] ade6-210 leu1-32 ura4-*
A9066	*cc2Δ*::*cc1 [Ch16-ade6-216] ars1*:*nmt41-GFP-Cnp1-NAT ade6-210 ura4-*

### Centromere plasmids and minichromosomes

Plasmids bearing centromere fragments contained a minimal *ars1* element to ensure efficient replication in *S*. *pombe*, in addition to selectable markers *sup3-5* (complements *ade6-704*), *ura4* ^*+*^ and KAN^R^. 8.5 kb of central domain DNA (*cc2* plus inner part of *imr2L* and *imr2R*) was cloned into the multiple cloning site as a *Sal*I-*Nco*I fragment to create pMcc2. Various sub-fragments of *cc2* (J-Q) were amplified by PCR and cloned into the multiple cloning site as *Bam*HI/*Bgl*II fragments. 5.6 kb of heterochromatin-forming outer repeat sequence was inserted adjacent to central domain sequences to test ability to form functional centromeres.

A plasmid, pMC28, bearing cc2, a KAN resistance marker and an inverted *ura4* sequence was constructed from pMcc2. Linearisation of the plasmid at *Not*I within the inverted *ura4* sequence allowed integration at *ura4* ^*+*^ located on the arm of Ch16-*m23*:*ura4* ^+^. Ch16-*m23*: *ura4* ^+^ is a derivative of Ch16, a 530 kb minichromosome, itself derived from Chromosome III [48]. It also bears the *ade6-216* allele which complements the *ade6-210* allele present on endogenous Chromosome III by interallelic complementation. Integration of linearised pMC28 on Ch16-m23:ura4 allowed selection on the counter-selective drug 5-fluoro-orotic acid and G418 (KAN). Cells that lost the Ch16-*m23*:*ura4*::*cc2-KAN* (abbreviated as Ch16-*cc2*) became red on limiting adenine and were sensitive to G418. For growth in liquid, cells containing Ch16-cc2 were grown in media lacking adenine.

### ChIP

ChIP was performed as previously described [84] using anti-CENP-A^cnp1^ antibody, anti CENP-C^Cnp3^ antibody, anti-CENP-K^Sim4^ antibody, anti-H3 antibody (ab1791; Abcam,), anti-H3K9me2 antibody (T. Urano) and anti-total RNA polymerase II (4F8; 61081, Active Motif), anti-Rpb1-Ser2P (3E10; 61083, Active Motif) and analysed by qPCR. Primers are listed in Table 2. P-values were calculated by standard t-test on 3 replicates between wild-type and mutant; p<0.05 was considered significant.

**Table 2:** Primers used in this study.

qact1 Fw	CCCAAATCCAACCGTGAGAAGATG
qact1 rev	CCAGAGTCCAAGACGATACCAGTG
qcnt1 fw	CAGACAATCGCATGGTACTATC
qcnt1 rev	AGGTGAAGCGTAAGTGAGTG
qpDF16-vect2 fw	ATAATACCGCGCCACATAGC
qpDF16-vect2 rev	CCAGAAACGCTGGTGAAAGT
qcnt2 L fw	GCATCTATTGTACTCTCTC
qcnt2 L rev	GAAGGATGGATATGCACGT
qcnt2 M fw	GTTAATTGCCATTCTTTGGCG
qcnt2 M rev	ATGACATGGCGTGGAAAGTC
qcnt2 N fw	CATTAAACAAACAACGGCACAC
qcnt2 N rev	TAAGCCAGCAAATTCCTTGAG
qcnt2 O fw	GACTATAACTAGACCACTCAG
qcnt2 O rev	CTAGATGAATACTCAAGAAAGC
qcnt2 P fw	CTGCATATTCGACATCTTGAG
qcnt2 P rev	AGCCTGTCCATCGCAAAAGG
CC2-F1 (J)	TACTACGGATCCCCCCATGGAATAAT
CC2-R1(J)	TACTACGTCGACCTCGAGATCTCAGTGCTCGCTTCTGTGTAA
CC2-F2 (K)	TACTACGGATCCGCGAGCACTGTTTACATCTAA
CC2-R2(K)	TACTACGTCGACCTCGAGATCTCGTCAAGTTTACAACTCGGT
CC2-F3(L)	TACTACGGATCCACCGAGTTGTAAACTTGACG
CC2-R3(L)	TACTACGTCGACCTCGAGATCTACAAATAGAATATGCTAACGC
CC2-F4(M)	TACTACGGATCCGCGTTAGCATATTCTATTTGTC
CC2-R4(M)	TACTACGTCGACCTCGAGATCTTGCGAGTTATGTGTATCTAC
CC2-F5(N)	TACTACGGATCCGTAGATACACATAACTCGCA
CC2-R5(N)	TACTACGTCGACCTCGAGATCTGGGGTCCTAGATTACGTCTT
CC2-F6(O)	TACTACGGATCCAATCTAGGACCCCTACGTTTT
CC2-R6(O)	TACTACGTCGACCTCGAGATCTCCCTTGGAAATACCTAGC
CC2-F7(P)	TACTACGGATCCGCTAGGTATTTCCAAGGGCTCC
CC2-R7(P)	TACTACGTCGACCTCGAGATCTTCCACCACAAATAGTTCAGC
CC2-F8(Q)	TACTACGGATCCGCTGAACTATTTGTGGTGGAC
CC2-R8(Q)	TACTACGTCGACCTCGAGATCTAAAGGGTATAAACGGCTATC
RegionL1-Lacz Fw	TACTACCTGCAGCTTGTCGCAAAACATAAAGC
RegionL1-Lacz rev	TACTACCTCGAGGTAAGTTTCTAAACAGGA
RegionL2-Lacz Fw	TACTACCTGCAGCGATAAACGTATGATTGCTTTCACC
RegionL2-Lacz rev	TACTACCTCGAGGAGAGAGTACAATAGATGC
RegionM1-Lacz Fw	TACTACCTGCAGCGTACCTTTTGACCTTTAAG
RegionM1-Lacz rev	TACTACGTCGACTCGAG CCGTATTTCACTTAAACGAG
RegionM2-Lacz Fw	TACTACCTGCAGTGCGAGTTATGTGTATCTAC
RegionM2-Lacz rev	TACTACGTCGACTCGAGCTCGTTTAAGTGAAATACGG
RegionO1-Lacz Fw	TACTACCTGCAGCCCTTGCCAGTAATGTGTAT
RegionO1-Lacz rev	TACTACCTCGAGCGCATTGAATTTTAGACTG
RegionO2-Lacz Fw	TACTACCTGCAGCATTCAAATTGCGTCAAC
RegionO2-Lacz rev	TACTACCTCGAGCCAAGTATATGTGTTCTACC
RegionP1-Lacz Fw	TACTACCTGCAGAACCAGATTTAATTAACGGCC
RegionP1-Lacz rev	TACTACCTCGAGGAAGCACTACTAAGATTAC
RegionP2-Lacz Fw	TACTACCTGCAGGTCGATTTTTCGAACTTTCTGC
RegionP2-Lacz rev	TACTACCTCGAGTTGGATTCGAAGGTCTTTAC
probe cc2 F	CGTGCACATTTGTGAAAAGG
probe cc2 rev	TCTCGCGATTAGTTTGTAAAG
probe cc1 F	CCATTTGCTAAGTTCGACTC
probe cc1 rev	CAGTATTTGTATCGTAGTGG

### Establishment assay

For the establishment assay, cells were transformed with minichromosomes (containing 5.6 kb of outer repeat sequence in addition to *cc2* sequences), by electroporation with ∼200 ng of DNA and plated on selective medium. Resultant colonies were replicated onto rich medium containing limiting adenine. The presence of pale pink/white colonies indicates establishment of a functional centromere on the minichromosome. Establishment efficiency is calculated as percentage of these colonies divided by the total number of transformants. Colonies were streaked on limiting adenine plates to confirm the presence of sectoring that is indicative of centromere function.

### Real-time PCR (qPCR)

Quantitative PCR reactions were carried out in 10 μl volume, with 5μl Light Cycler 480 SybrGreen Master Mix (Roche), 0.5μl each primer (10 μM) and 3μl ChIP or total template. The data were analysed using Light Cycler 480 Software 1.5 (Roche).

### 5’RACE-PCR and RT-PCR

5’RACE-PCR was performed as previously described [37]. In brief, RNA was isolated with RNeasy mini/midi kit (Qiagen) according to the manufacturer’s protocol. Poly(A) containing RNA was purified from 500 μg of total RNA by affinity purification with biotinylated oligo-dT using PolyATtract mRNA Isolation Systems (Promega). 5’RACE PCR was performed using SMARTer 5’/3’ RACE (Clontech) according to the manufacturer’s protocol. PCR products were then run on 1% agarose gel, purified and cloned into pGEM-T Easy vector (Promega) and subsequently sequenced. Reverse transcription reaction for 5’RACE and qRT-PCR was performed using Superscript III Reverse Transcriptase (Invitrogen) using RNA extracted from 3 independent colonies. For qRT-PCR, transcript levels were normalized over gDNA to take into account differences in copy number between plasmids and normalized relative to *act1* ^*+*^.

### LacZ assay

LacZ assay was performed as described [85]. pREP81X-LacZ was digested with *Xho*I and *Pst*I and the nmt81 promoter upstream of LacZ replaced with sequences from centromere 2. Plasmids were transformed into wild-type and grown on minimal medium (n = 3).

### Southern analysis

DNA was extracted as previously described [83]. The DNA was digested with *Bgl*II/*Spe*I or *Sph*I/*Spe*I, run on a 1% agarose gel, blotted on nylon membrane (Hybond N, Amersham) and UV-crosslinked. The membrane was hybridized with DNA probes specific for central domain 1 or central domain 2. To make the probes, PCR products were used as template in the labelling reaction using High Prime (Roche). Primers sequences are listed in Table 2.

### Western analysis

Western analysis was performed as described previously using anti-GFP antibody (Roche) and anti-H3 antibody (ab1794-abcam) [86]. The intensities of GFP and H3 signals were acquired using LICOR Odyssey Infrared Imaging System software (Li-COR Bioscience).

## Supporting Information

S1 Fig Replacement of central core 2 sequence from endogenous centromere 2 with central core 1 DNA. (A) Schematic representation of endogenous centromere 1 (*cnt1*), centromere 2 (*cnt2*) and central core 2 deletion (*cc2Δ*). The probes on *cc2* (grey) and *cc1* (black) used for Southern analysis and the expected size of DNA fragments following restriction enzyme digestion are indicated. (B) Genomic DNA was extracted from wild-type cells and *cc2*Δ strain. DNA was digested with *Bgl*II and *Spe*I and the Southern hybridised with the 2.8 kb *cc1/3* probe. (C) Genomic DNA was extracted from wild-type cells and the *cc2*Δ strain. The DNA was digested with *Sph*I and *Spe*I and the Southern hybridised with the 2.8 kb *cc2* probe. The absence of the band for cc2 indicates its deletion from the endogenous *cen2* locus. (D) Spotting assay of wild-type or *clr4Δ* cells containing centromere 2 sequence at the endogenous centromere (*cc2*+) or wild-type cells with *cc2* sequence replaced by *cc1* (*cc2*Δ). YES: complete medium. Phloxin B stains dead cells red. Cells with impaired centromere function (e.g. *clr4*Δ) show sensitivity to the microtubule destabilizing drug thiabendazole (TBZ), while *cc2* ^+^ and *cc2*Δ are able to grow in the presence of the drug TBZ suggesting that *cc2*Δ does not affect centromere function.
(EPS)

S2 Fig Plasmids containing a combination of the LM and OP fragments are able to CENP-A^Cnp1^. (A) ChIP analysis of CENP-A^Cnp1^ levels of wild-type (wt-CENP-A^Cnp1^) and cells overexpressing CENP-A^Cnp1^ (hi-CENP-A^Cnp1^) transformed with a plasmid containing a combination of LM and OP fragment (OP-LM-OP). (B) Establishment assay using plasmids containing 5.6 kb of heterochromatin-forming outer repeat element flanking a full length *cc2* (pH-cc2), three tandem repeats of the LM region (pH-3xLM), three tandem repeats of the OP region (pH-3xOP) or one copy of the LM fragment adjacent to two copies of OP (pH-LM-2xOP). pH-cc2, pH-3xLM, pH-3xOP and pH-LM-2xOP were transformed in wild-type cells and the percentage of transformants that contained plasmids with centromere function was assessed by replica plating onto limiting adenine medium. White colonies indicate formation of functional centromeres.
(EPS)

S3 Fig RNA polymerase II is present on central core sequences when CENP-A^Cnp1^ is not established. ChIP of RNA polymerase II (RNAPII) on the *p3xLM* (A) and *p3xOP* (B) plasmids transformed into wild-type cells. Positions of the PCR products quantified for each plasmid are indicated. (C) ChIP of RNA polymerase II (RNAPII) on the pMcc2 plasmid transformed into wild-type (wt-CENP-A^Cnp1^) and cells overexpressing CENP-A^Cnp1^ (hi-CENP-A^Cnp1^). The enrichment is shown as relative to *act1* ^+^ (n = 3).
(EPS)

S4 Fig Identification of Transcriptional Start Sites (TSS) within the 2 kb LM and OP regions. (A) Example of RT-PCR products obtained from the 5’RACE reaction. Generally, multiple products were identified for the centromeric regions, while one single product was identified from the *act1* gene. *(B)* Sequences of TSSs detected by 5’RACE in LM and OP regions. (C) Analysis of promoter activity of the ∼200 bp regions from *cc2*. Region-M1 and region-M2 were placed upstream of a LacZ reporter gene. The levels of LacZ expression were assessed by measuring absorbance at 420 nm of cell lysates incubated with 2-Nitrophenyl-β-D-galactopyranoside (ONPG). region-M2 inv: region cloned inverted; region-M2 mut: point mutations inserted in the M2sequence. nmt81: positive control with *nmt81* promoter (n = 3).
(EPS)

S5 Fig Lack of *S*. *pombe* TFIIS increases CENP-A^Cnp1^ deposition at pMcc2 in hi-CENP-A^Cnp1^ cells. Enrichment levels of CENP-A^Cnp1^ at the endogenous centromeres (*cc1/cc3*) and at the pMcc2 plasmid transformed in wild-type cells (wt), in cells expressing high levels of CENP-A^Cnp1^ (hi-CENP-A^Cnp1^) and in hi-CENP-A^Cnp1^ in which the transcription factor TFIIS (Tfs1) is deleted (*tfs1Δ*+hi-CENP-A^Cnp1^) (n = 3).
(EPS)

S6 Fig Lack of Ubp3 allows establishment of CENP-A^Cnp1^chromatin on p3xLM but not p3xOP. ChIP for CENP-A^Cnp1^ on a p3xLM and p3xOP plasmids transformed into wild-type (wt) or a strain deleted for Ubp3 (*ubp3Δ*). CENP-A^Cnp1^ levels are shown relative to *cc1/3*.
(EPS)

S7 Fig Deletion of *ubp3* and *tfs1* does not affect CENP-A^Cnp1^ protein levels. Western analysis and protein levels comparison between wild-type (wt), *ubp3Δ* and *tfs1Δ* of endogenous N-terminal tagged GFP-CENP-A^Cnp1^ and H3. Ponceau staining as internal loading control.
(EPS)

S8 Fig Cells lacking Ubp3 show reduced levels of CENP-A^Cnp1^ at endogenous centromeres. (A) ChIP for CENP-A^Cnp1^, CENP-C^Cnp3^ and CENP-K^Sim4^ levels at endogenous centromere *cc1/3* in wild-type (wt), *tfs1Δ* and *ubp3Δ*. *p<0*.*05* is indicated with an asterisk (n = 3). (B) ChIP for CENP-C^Cnp3^ and CENP-K^Sim4^ levels at pMcc2 plasmid transformed into wt, *tfs1*Δ and *ubp3*Δ. *p<0*.*05* is indicated with an asterisk (n = 3).
(EPS)

S9 Fig CENP-A^Cnp1^ deposition on pcc2 plasmid is not dependent on H3K9me2 in the *tfs1Δ* and *ubp3Δ* mutants. (A) ChIP for H3K9me2 (A) and CENP-A^Cnp1^ (B) at *act1* ^+^ and at pMcc2 plasmid transformed in wt, *clr4Δ*, *ubp3Δ* and *ubp3Δclr4Δ*. CENP-A^Cnp1^ is deposited on pcc2 in *ubp3* regardless of whether Clr4 is present or absent. (C) ChIP for H3K9me2 at *act1* ^+^ and on the pcc2 plasmid transformed into wt, *tfs1Δ* and *tfs1Δclr4Δ* cells. The absence of H3K9me2 on the plasmid suggests that heterochromatinisation is not responsible for CENP-A^Cnp1^ deposition. (D) CENP-A^Cnp1^ levels on *act1* ^+^ and at the pcc2 plasmid in wt, *tfs1Δ* and *tfs1Δclr4Δ*. The absence of the methyltranferase Clr4 does not affect CENP-A^Cnp1^ deposition on pMcc2.
(EPS)
